# Dynamics of muscle fibre growth during postnatal mouse development

**DOI:** 10.1186/1471-213X-10-21

**Published:** 2010-02-22

**Authors:** Robert B White, Anne-Sophie Biérinx, Viola F Gnocchi, Peter S Zammit

**Affiliations:** 1 King's College London, Randall Division of Cell and Molecular Biophysics, Guy's Campus, London SE1 1UL, UK

## Abstract

**Background:**

Postnatal growth in mouse is rapid, with total skeletal muscle mass increasing several-fold in the first few weeks. Muscle growth can be achieved by either an increase in muscle fibre number or an increase in the size of individual myofibres, or a combination of both. Where myofibre hypertrophy during growth requires the addition of new myonuclei, these are supplied by muscle satellite cells, the resident stem cells of skeletal muscle.

**Results:**

Here, we report on the dynamics of postnatal myofibre growth in the mouse extensor digitorum longus (EDL) muscle, which is essentially composed of fast type II fibres in adult. We found that there was no net gain in myofibre number in the EDL between P7 and P56 (adulthood). However, myofibre cross-sectional area increased by 7.6-fold, and length by 1.9-fold between these ages, resulting in an increase in total myofibre volume of 14.1-fold: showing the extent of myofibre hypertrophy during the postnatal period. To determine how the number of myonuclei changes during this period of intense muscle fibre hypertrophy, we used two complementary mouse models: *3F-nlacZ-E *mice express *nlacZ *only in myonuclei, while *Myf5*^*nlacZ*/+ ^mice have β-galactosidase activity in satellite cells. There was a ~5-fold increase in myonuclear number per myofibre between P3 and P21. Thus myofibre hypertrophy is initially accompanied by a significant addition of myonuclei. Despite this, the estimated myonuclear domain still doubled between P7 and P21 to 9.2 × 10^3 ^μm^3^. There was no further addition of myonuclei from P21, but myofibre volume continued to increase, resulting in an estimated ~3-fold expansion of the myonuclear domain to 26.5 × 10^3 ^μm^3 ^by P56. We also used our two mouse models to determine the number of satellite cells per myofibre during postnatal growth. Satellite cell number in EDL was initially ~14 satellite cells per myofibre at P7, but then fell to reach the adult level of ~5 by P21.

**Conclusions:**

Postnatal fast muscle fibre type growth is divided into distinct phases in mouse EDL: myofibre hypertrophy is initially supported by a rapid increase in the number of myonuclei, but nuclear addition stops around P21. Since the significant myofibre hypertrophy from P21 to adulthood occurs without the net addition of new myonuclei, a considerable expansion of the myonuclear domain results. Satellite cell numbers are initially stable, but then decrease to reach the adult level by P21. Thus the adult number of both myonuclei and satellite cells is already established by three weeks of postnatal growth in mouse.

## Background

The first 3 weeks of postnatal life in mouse is a period of intense growth, with body weight increasing 7-8 fold, half of which is accounted for by the increase in skeletal muscle [1]. Postnatal muscle growth is achieved by an increase in number (hyperplasia) and size (hypertrophy) of myofibres in rat [2,3], but mainly by hypertrophy of myofibres in mouse [4]. The extensor digitorum longus (EDL) muscle of the crural hind limb has been well studied. In rat EDL, there is an increase in myonuclei until at least 100 days of age [5]. In mouse however, the EDL achieves its full complement of myofibres between embryonic day (E) 18 and birth [4]. There is a lack of information about myonuclei accretion in the early postnatal period, but little addition of myonuclei from postnatal day (P) 14 onwards has been reported, although the mouse EDL increases rapidly in size from this point; with a 3.5 fold increase in weight between 2-17 weeks of age [4].

The new myonuclei required during postnatal muscle growth are provided by muscle satellite cells [6-8], which occupy a distinct niche on the surface of the myofibre, beneath the surrounding basal lamina [9,10]. Cells in this location can first be identified in foetal mouse muscle around E16.5, after the formation of the basal lamina [11]. At birth, muscle satellite cells have been reported to comprise 30-35% of sublaminal nuclei in mouse peroneus longus muscle, and 28% of sublaminal nuclei in mouse lumbrical muscle at P7 [12,13]. Many of these satellite cells (~80%) are proliferating in both growing mouse and rat [14,15] and labelling of DNA-synthesis is greatest before P21 in mouse, after which it declines sharply [16]. The majority of satellite cells become mitotically quiescent in mature muscle [17] with only <1% still incorporating label during cell division in 6 to 8 week old mice [4,18]. The proportion of satellite cells also gradually falls as growth proceeds [12,13,19], for example dropping from 11% at P14 in mouse EDL to 3% at 17 weeks of age, with absolute numbers also falling by a similar amount [4]. Thus most satellite cells are dividing to provide new myonuclei during postnatal growth, before a population of uniformly mitotically quiescent satellite cells is established.

There is however, a lack of data in mouse from the first two postnatal weeks, effectively missing, what studies suggest, to be the most dynamic period of postnatal growth. By using isolated myofibres from genetically modified mice [20-22] at different ages, it is possible to track changes in myofibre dimensions, and relate this to the number of myonuclei and satellite cells. For this study, we chose to examine the EDL as it is possible to remove it intact from relatively young postnatal mice to section or prepare myofibres from. It has a near uniform fast fibre type composition in adult, with <1% slow fibres [23], and this relative distribution of fast and slow fibres is established early, and does not change during postnatal growth [24,25], reducing any impact of fibre type differences on myonuclear or satellite cell number [26]. In addition, it has been the subject of similar studies in the past e.g. Ontell et al. [4].

Here, we report that total myofibre number in EDL remained unchanged between P7 and P56, while myofibre cross-sectional area increased by 7.6-fold and length by 1.9-fold, resulting in an expansion in myofibre volume of 14.1-fold. Using two independent mouse models, we found that myonuclear accretion in EDL myofibres occurred only within the first three weeks postnatally, with a 4.9-fold increase between P3 and P21. The number of satellite cells was initially similar to the daily rate of myonuclear addition, with ~14 satellite cells per myofibre until P9, when the calculated mean myonuclear accretion rate is 13.6 nuclei per day. Between P14 and 21, the addition of myonuclei decreased to 5.6 nuclei per day, while the available satellite cells fell from ~10, to ~5, per myofibre. Despite the addition of new myonuclei, the greater proportional increase in myofibre volume meant that the myonuclear domain doubled between P7 and P21 to an estimated 9.2 × 10^3 ^μm^3^. The adult number of myonuclei and satellite cells is established by three weeks of postnatal development. Thus the calculated mean myofibre volume increases 3-fold between P21 and P56 (adulthood), without the net addition of new myonuclei, resulting in a further significant expansion of the myonuclear domain in fast myofibres, to an estimated 26.5 × 10^3 ^μm^3^.

## Results

### Total myofibre number does not change during postnatal growth of EDL muscle

**Figure 1:**
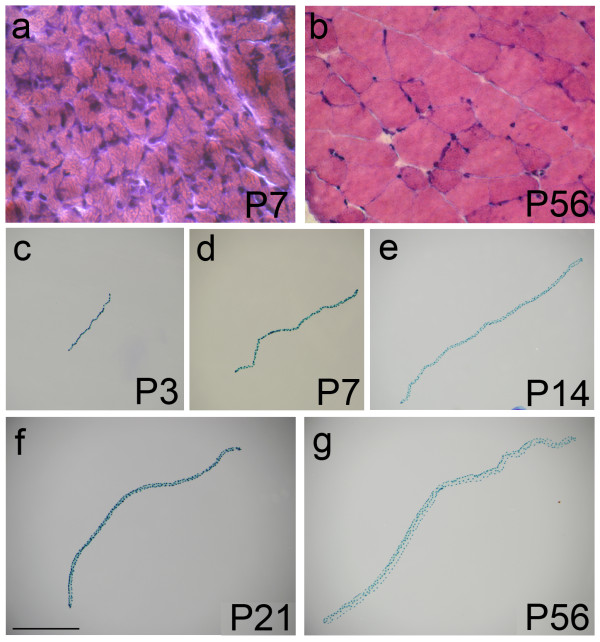
**EDL myofibres from growing and adult mice**. Entire EDL muscles from P3, P7 (a), P14, P21 and P56 (b) *Myf5*^*nlacZ*/+ ^mice were cryosectioned and mid-belly sections stained with haematoxylin and eosin to determine total myofibre content and myofibre cross-sectional area. EDL myofibres were isolated from *3F-nlacZ-E *mice at P3 (c), P7 (d), P14 (e), P21 (f) and P56 (g), fixed and incubated in X-gal solution to reveal the β-galactosidase activity of myonuclei by the presence of the blue reaction product. Images of representative myofibres were all taken at the same magnification and show that there is a 4.5-fold increase in length between P3 and adulthood (P56). Myonuclei appear to be uniformly distributed along the length of a myofibre at each age examined (c-g). Scale bar equals 100 μm for (a and b) and 1000 μm for (c-g).

Muscle growth can be achieved by an increase in myofibre number and/or an expansion of individual myofibres. To first examine if hyperplasia of the EDL contributed to muscle growth during the postnatal period in mouse, we counted the number of myofibres contained in the mid-belly section of muscles dissected from P7, P14, P21 and P56 (adulthood) mice on haematoxylin and eosin stained (Figure 1a and 1b), or laminin-immunostained, cryosections (Table 1). This analysis showed that there was no change in EDL myofibre number between P7 and P56 (Table 1) (ANOVA: F = 1.0; *p *= 0.42 - the fall between 3 and 8 weeks is not significant, ANOVA with Tukey HSD post hoc testing: *p *= 0.50).

**Table 1:** Number of myofibres and satellite cells in EDL muscle sections

*Age (days)*	P7	P14	P21	P56
*Myofibres per EDL cross-section*	**1336** ±68 (3)	**1343** ±105 (5)	**1346** ±30 (3)	**1147** ±71 (3)

*Myofibre cross-section area (μm*^*2*^)	**179.2** ±4.1 (3)	330.0* ±6.6 (5)	550.1* ±10.0 (3)	1363.8* ±33.7 (3)

*Satellite cells per EDL cross-section*	**53.7** ±14.3 (3)	13.3* ±2.4 (5)	**15.7** ±5.4 (3)	**17.7** ±0.3 (3)

*Satellite cells per myofibre*	**0.040**	**0.010**	**0.012**	**0.015**

Myofibres and satellite cells (identified by X-gal staining) were counted per cross section at mid-belly level, and cross-sectional area was measured from at least 100 myofibres, from each of three *Myf5*^*nlacZ*/+ ^mice at each age. Data shown are mean ± SEM (n: mice). Asterisk indicates significant difference from preceding value (ANOVA with Tukey HSD post-hoc testing; p < 0.01).

### Myofibre dimensions increase significantly during postnatal EDL growth

**Figure 2:**
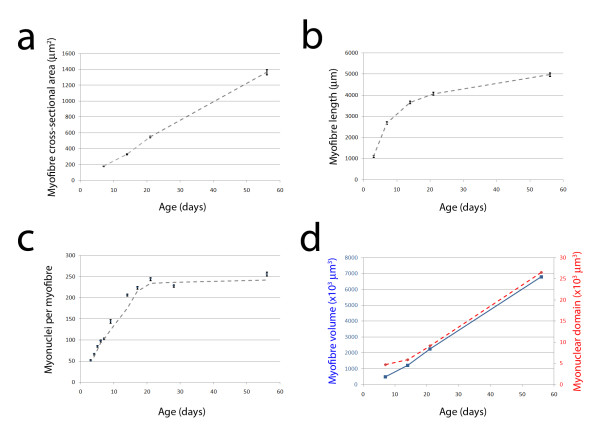
**Postnatal growth dynamics of EDL myofibres**. Measuring myofibre cross-sectional area from mid-belly EDL muscle sections showed that there was a significant (ANOVA: *p *< 0.0001; F = 850.6) and highly linear (*R*^2 ^= 1.0) increase with age (a), which was accompanied by a significant increase in the length of isolated EDL myofibres (b). Myonuclei were counted on isolated EDL myofibres from *Myf5*^*nlacZ*/+ ^and/or *3F-nlacZ-E *mice and there was a 4-fold increase in myonuclei number per myofibre between P3 and P14 from ~50 to ~200 (c). The adult complement of myonuclei (~250) was reached by P21. Mean total myofibre volume was calculated by multiplying mean length by mean cross-sectional area and increased in a linear fashion between P7 to P56 (blue line - d). By then dividing mean total myofibre volume by the mean number of myonuclei per myofibre, we estimated myonuclear domain during postnatal development (dashed red line - d). Data shown are mean ± SEM, except for (d), which is mean; trend line in (c) depicts the moving average [(a) n > 100 myofibres from each of at least 3 mice at each age; (b) n > 19 myofibres from each of at least 3 mice at each age; (c) n > 30 myofibres from each of at least 2 mice at each age, see Tables 1 and 2 for data sets].

To assess myofibre hypertrophy, we first determined cross-sectional area of myofibres from mid-belly muscle sections of EDL between P7 and P56 (Figure 1a and 1b). We found that cross-sectional area increased significantly at each time-point analysed (ANOVA: *p *< 0.0001; F = 850) in a linear manner (*R*^2 ^= 0.99 - Figure 2a), from 179.2 ± 4.1 μm^2 ^at P7, to 1363.8 ± 33.7 μm^2 ^at P56 (Table 1).

**Table 2:** Summary of the growth dynamics of EDL myofibres

*Age (days)*	P3	P7	P14	P21	P56
*Myofibre length (μm)*	**1104.5** ±43.8 (20)	2688.6* ±66.0 (31)	3663.0* ±57.8 (36)	4069.8* ±70.7 (42)	4977.8* ±83.9 (31)

*Sarcomere length (μm)*	**-**	**2.16** ±0.08 (764)	**-**	**2.15** ±0.07 (947)	**2.16** ±0.07 (969)

*Myonuclei per myofibre*	**51.9** ±1.4 (71)	102.8* ±2.0 (61)	206.7* ±2.4 (204)	244.5* ±3.5 (111)	**256.1** ±4.7 (78)

*Myonuclei per Unit length(100 μm)*	4.7	3.7	5.6	6	5.1

*Satellite cells per myofibre*	**-**	**14.3** ±0.7 (61)	9.4* ±0.4 (204)	4.5* ±0.3 (111)	**5.8** ±0.3 (78)

Data are mean ± SEM (n: number of myofibres) from 3 × *3F-nlacZ-E *mice per age for length measurements and at least 3 each of *Myf5*^*nlacZ*/+ ^and *3F-nlacZ-E *mice per age for myonuclear and satellite cells counts. For sarcomere length, data are mean ± SEM (n: number of sarcomeres) from three mice at each age. Asterisk indicates significant difference from preceding value (ANOVA with Tukey HSD post-hoc testing; p < 0.01).

To determine the length of muscle fibres, we used collagenase digestion to isolate viable myofibres from EDL muscles of *3F-nlacZ-E *mice at P3, P7, P14, P21, and P56 (Figure 1c-g). Individual intact myofibres were fixed and measured and we found that the mean length increased from 1105 ± 44 μm at P3, to 2689 ± 66 μm at P7 and 3663 ± 58 μm by P14 (Table 2). Adulthood was considered reached by P56, at which time the mean myofibre length was 4978 ± 84 μm, an approximate 4.5-fold increase from P3 (Table 2). Myofibre elongation over this period was logarithmic (*R*^2 ^= 0.97 - Figure 2b). To examine if changes in myofibre length were influenced by the relaxation state at different ages, we also measured sarcomere length of isolated myofibres at P7, P21, and P56 (Table 2). Sarcomere lengths were close to reported figures of living mouse EDL myofibres (e.g. 2.35 μm [27]), but importantly, were not significantly different between the ages examined (ANOVA: *p *= 0.5669; F = 0.5676).

### Myonuclear accretion in EDL myofibres is complete by P21

**Figure 3:**
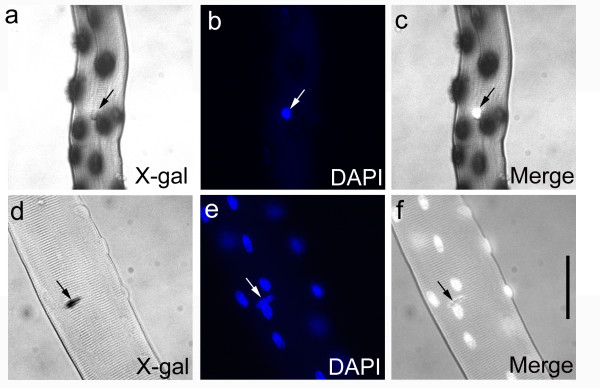
**Identification of myonuclei and satellite cells from growing *3F-nlacZ-E *and *Myf5 ***^***nlacZ*/+ **^**mice**. Myofibres were isolated from P14 mice, fixed and incubated in X-gal and DAPI. Myonuclei are revealed in *3F-nlacZ-E *myofibres by β-galactosidase activity producing a blue reaction product (a), whereas satellite cells are visible as DAPI-positive nuclei, since the X-gal reaction product obscures the DAPI fluorescence in myonuclei (b). This enables clear discrimination between myonuclei and satellite cells, as seen in the merged image (c). By contrast, in *Myf5*^*nlacZ*/+ ^mice, satellite cells are identified by their β-galactosidase activity on isolated myofibres (d), whereas myonuclei are DAPI-positive nuclei that do not contain the blue X-gal reaction product (e and f). Scale bar equals 50 μm.

To determine both the rate of myonuclear addition and the point at which the myofibre attains its full adult complement of myonuclei, we used two complementary mouse models. The *3F-nlacZ-E *mouse [21,28] contains a transgene in which elements of the *myosin light chain 3F *locus drive a *nlacZ *reporter gene in all fast muscle myonuclei (Figure 1c-g and 3a-c). The *Myf5*^*nlacZ*/+ ^mouse [20] has β-galactosidase activity in satellite cells from the *nlacZ*-targeted *Myf5 *allele [21], allowing the number of myonuclei to be determined by counting DAPI-positive/β-galactosidase-negative nuclei (Figure 3d-f). Intact isolated myofibres were fixed in paraformaldehyde, incubated in X-gal and mounted with DAPI. The number of myonuclei per myofibre at P3, P7, P14, P21, and P56 counted in these two independent mouse models was not significantly different at each age (ANOVA: *p *= 0.79; F = 0.07), and so the data were combined (Table 2). We did not formally analyse the distribution of myonuclei along the length of a myofibre, but they appeared to be uniformly distributed at all ages examined (Figure 1c-g).

Analysis of myonuclei per muscle fibre between P3 and P56 (Figure 2c) revealed that EDL myofibre growth goes through distinct phases. During the first two postnatal weeks, myofibres accumulate 13.6 nuclei per day (*R*^2 ^= 0.98), with the mean myonuclear capacity of EDL myofibres undergoing two complete doublings between P3 (~52 myonuclei) and P14 (~207 myonuclei). Indeed they reach ~81% of the adult myonuclear number by P14, from just ~20% at P3 (Table 2). In the third postnatal week, myonuclear accretion per myofibre decreased to 5.6 nuclei per day (*R*^2 ^= 1.0). No significant difference in the number of myonuclei per myofibre was observed from P21 onwards, with mice at the oldest age examined of 10 months having a mean ± SEM of 259.1 ± 9.2 myonuclei (10-15 myofibres from each of three mice) (ANOVA with Tukey HSD post hoc testing: P21-P56: *p *= 0.81; P21-10 months: *p *= 0.39). Therefore, EDL myofibre hypertrophic growth after P21 is without the addition of new myonuclei (Figure 2, Table 1 and 2).

### Myonuclear domain increases throughout postnatal development

Having both measured the mean myofibre length and counted the total number of myonuclei per myofibre from the same mice during postnatal growth, we could determine the number of myonuclei per unit myofibre length (Table 2). This showed that there was no great change between P7 and P56, even though the myofibres increased in length by 1.9-fold over this period. However, between these ages the radial growth of the myofibres resulted in a 7.6-fold increase in cross-sectional area (Table 1). Although measured from different cohorts of age-matched mice, using the myofibre length and cross-sectional area data allowed us to calculate a mean myofibre volume at each age examined (Figure 2d). Mean total myofibre volume increased from 481.8 × 10^3 ^μm^3 ^at P7 to 6788.7 × 10^3 ^μm^3 ^at P56 (Table 3).

**Table 3:** Myofibre volume and myonuclear domain during growth of EDL myofibres

*Age (days)*	P7	P14	P21	P56
*Myofibre volume (*×10^3 ^μm^3^)	**481.8**	**1208.8**	**2238.8**	**6788.7**

*Myonuclear domain (*×10^3 ^μm^3^)	**4.7**	**5.9**	**9.2**	**26.5**

Mean total myofibre volume was calculated from multiplying mean myofibre length by mean myofibre cross-sectional area at each age. Myonuclear domain was derived by then dividing mean total myofibre volume by the mean number of myonuclei per myofibre at each age.

To obtain an estimate of myonuclear domain that took into account both radial and lengthwise myofibre growth, we then divided mean myofibre volume by the mean number of myonuclei per myofibre at each age (Table 3 and Figure 2d). Despite the continued addition of myonuclei up to P21, the increase in myofibre volume was greater, so that the estimated myonuclear domain virtually doubled from 4.7 × 10^3 ^μm^3 ^at P7, to 9.2 × 10^3 ^μm^3 ^at P21. From P21 there was a 3-fold increase in myofibre volume without the net addition of further myonuclei, meaning that the myonuclear domain also expanded an estimated ~3-fold to 26.5 × 10^3 ^μm^3 ^at P56.

### Satellite cell number falls during postnatal growth

Having shown that myofibre length, cross-sectional area and number of myonuclei increased significantly during the first three postnatal weeks, we next examined the number of satellite cells over this period, since they are the source of postnatal myonuclei [8]. To identify satellite cells on isolated myofibres, we used our two mouse models that either positively or negatively mark satellite cells (Figure 3). As mentioned above, satellite cells in *Myf5*^*nlacZ*/+ ^mice are identified directly by the expression of nuclear-localised β-galactosidase from the targeted *Myf5 *allele [21]. We also used the *3F-nlacZ-E *transgenic mouse to quantify satellite cells on isolated myofibres, since β-galactosidase-negative nuclei are those of satellite cells, providing a means to identify the entire population, regardless of antigen expression [21].

**Figure 4:**
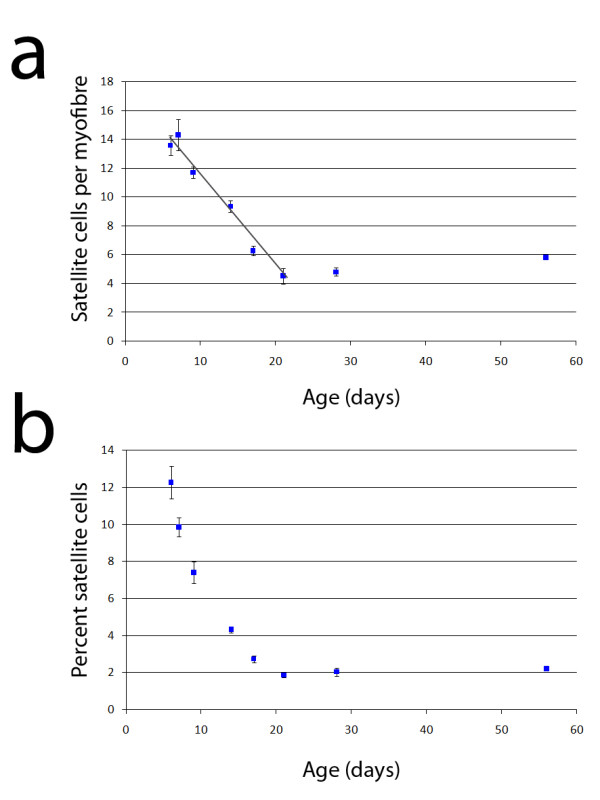
**Satellite cell number steadily decreases from P6, to reach the adult level by P21**. Satellite cells were identified and counted on isolated *Myf5*^*nlacZ*/+ ^and *3F-nlacZ-E *myofibres and pooled at each age to give a mean ± SEM (a). The number of satellite cells per myofibre decreased by 0.66 satellite cells per day (*R*^2 ^= 0.98) to reach the adult level by P21, when it remained unchanged until 10 months, our oldest age analysed. The same data with satellite cells expressed as a percentage of total myofibre nuclei (myonuclei plus satellite cells) (b). Due to the rapidly increasing myonuclear number, there is an exaggerated fall from P6 until P21. Data shown are mean ± SEM (n > 30 myofibres from at least 6 mice at P7, P14, P21 and P56, and at least 2 mice per age at all other stages).

At P7, each myofibre had a mean of ~14 satellite cells (Table 2 and Figure 4a), which then fell by 0.7 cells per myofibre each day (*R*^2 ^= 0.97) to reach ~5 by P21, a fall of ~68% between P7 and P21. Satellite cells on EDL myofibres reach their adult level by P21, with no significant change thereafter (Figure 4a - ANOVA with Tukey HSD post hoc testing: P21: *p *= 0.96; P56: *p *= 0.93), until at least the latest age examined of 10 months. Thus before P9, the number of satellite cells per myofibre is similar to the daily nuclear accretion rate: ~13 satellite cells per myofibre when the mean rate of myonuclear addition is 13.6 nuclei per day. Between P14 and P21, the myonuclear accumulation rate decreases to 5.5 nuclei per day when the available mean satellite cell number decreases to ~5 per myofibre.

We also expressed satellite cell numbers as a percentage of total nuclei per myofibre (Figure 4b), to enable comparison with other studies. This equated to 9.86% at P7, 7.4% at P9 and falling to 4.6% at P14. However presenting the data this way exaggerates the rate of decline, since the denominator is not a constant, as mean myonuclear number increased ~2 fold over this period (Table 2 and Figure 2c).

Most studies examining satellite cell numbers during postnatal growth have counted cells on muscle sections (e.g. Ontell et al. [4]). Therefore, we also determined satellite cell number in entire mid-belly EDL sections from P7, P14, P21 and P56 mice, as an independent method to measure the dynamics of satellite cells in growing muscle. We used β-galactosidase activity from the targeted *Myf5 *allele in *Myf5*^*nlacZ*/+ ^mice to identify and count satellite cells. Mean satellite cell number per EDL cross-section was 53.7 ± 14.3 at P7, falling to 13.3 ± 2.4 at P14, which was not significantly different from the number at P21 and P56 (Table 1).

## Discussion

During the first three postnatal weeks in mouse, body mass and total muscle mass both increase almost 3-fold, and fibre length within the tibialis anterior muscle increases by more than 4-fold [1]. Muscles can grow by an increase in total myofibre number and/or an increase in the size of individual myofibres. We found that the total number of myofibres in the mouse EDL did not increase from P7, in agreement with previous studies that myofibre number is set around birth in many muscles [4,24]. Therefore, postnatal growth occurs by hypertrophy of existing myofibres. Indeed, between P7 and P56, myofibre cross-sectional area increased by 7.5-fold and length by 1.9-fold, together producing a mean total volume rise of 14.1-fold over this period. Such hypertrophy significantly contributes to the 3.5-fold increase in mouse EDL weight reported between 2-17 weeks of age [4].

We also counted the number of myonuclei per myofibre using two independent mouse models and found that myonuclear addition only occurred during the first three postnatal weeks. We did not observe any regions of obvious higher myonuclear density during postnatal growth, as may have been expected if myonuclei were added preferentially to a particular region of the myofibre. Myonuclear addition occurs mainly at the ends of myotubes during rat foetal developmental myogenesis [29] and possibly in growing chicken muscle, where a high myonuclear density has been reported at myotendinous junctions [30,31]. However, there is no clear evidence that this also happens during postnatal growth or regeneration in mammals [15,32], even though sarcomeres are serially added to the ends of mammalian myofibres during growth, and in mature muscles undergoing adaptation [33].

The first two weeks are the most active period for the addition of new myonuclei, with myonuclear number doubling twice from P3 to P14. The number of myonuclei increases from ~20% of the adult total, to ~81% by P14, by the addition of over 13 myonuclei per myofibre each day. Since muscle fibres are syncytia, each myonucleus supports a certain volume of cytoplasm in the myofibre - the myonuclear domain. Despite the addition of these new myonuclei between P7 and P14, the myofibre volume increased at a greater rate, so that the estimated myonuclear domain still expanded 1.2-fold. Between P14 and P21, accumulation falls to 5.5 myonuclei per day, with the full adult complement being reached by P21. The greater increase in myofibre volume though, again results in myonuclear domain increasing by a further 1.6-fold. Thus, although the myofibre hypertrophy that occurs during early postnatal growth (P7-P21) is clearly supported by significant myonuclear accretion, the myonuclear domain still doubles due to the greater increase in myofibre volume. These observations refine the electron microscopy studies of Ontell and colleagues [4] in mouse EDL, which indicated that the adult number of myonuclei was attained by their first assay point at P14, not changing when next determined at P28 or P56. Our data shows that addition of new myonuclei continues for approximately another week, ending by P21.

Myofibres do not stop growing at P21 however, when myonuclear accretion ceases; we found that myofibres increased in cross-sectional area by almost 2.5-fold and length by 1.2-fold between P21 and P56, accounting for an approximate 3-fold expansion in myofibre volume. Ontell et al. [4] reported that while mouse EDL increases in mass by 25%, cross-sectional area only increased by ~20% between 4 and 8 weeks after birth, so presumably, most of the increase in cross-sectional area occurring after P21, happens in the following week. Since this significant 3-fold increase in myofibre hypertrophy after P21 is entirely orchestrated without the addition of significant numbers of myonuclei, the myonuclear domain must also expand, in fact rising a further 2.9-fold to 26.5 × 10^3 ^μm^3^.

The estimated myonuclear domain expanded ~5.7-fold from P7 to P56, consistent with reported increases in growing rat between P14 and P28 in diaphragm [34] and the 4-fold increase in soleus between P4 and P56 [35]. At P56, our calculated myonuclear domain is 26.5 × 10^3 ^μm^3 ^for entire intact myofibres. Liu et al. [36] used myofibre fragments from 6 month old mouse EDL and calculated myonuclear domains for IIx of 46.1 × 10^3 ^μm^3^, IIb of 48.5 × 10^3 ^μm^3 ^and IIx/b of 31.5 × 10^3 ^μm^3^, which, we calculate, gives a mean of 44.9 × 10^3 ^μm^3 ^for EDL muscle fibres. Therefore, there is an approximate 70% increase in myonuclear domain between 8 weeks (our data) and 6 months of age [36] in mouse. Since we found that the number of myonuclei does not change between 8 weeks and 10 months, then the myonuclear domain can only expand with any further growth; indeed myonuclear domain has been reported to be 88% greater at 14 months than at 8 weeks in mouse EDL [37]. It is worth noting that mice lacking the TGFβ-family member *myostatin *(a strong inhibitor of myogenesis) have significantly larger EDL myofibres (42.8% greater cross-sectional area and 5.9% longer at 8 weeks of age) but less myonuclei per myofibre, showing that myonuclear domain is highly flexible during development [38]. Certainly in adult human muscle, training-induced muscle hypertrophy can occur with an expansion of the myonuclear domain [39,40]. The considerable enlargement of myonuclear domain after P21 probably results from concomitant myonuclear maturation, increased protein synthesis and completion of protein isoform switching, including neonatal to adult MyHC isoforms [30,41,42].

We did not assess muscle fibre type in our study. Myonuclear and satellite cell numbers differ between slow and fast fibre types, with slow generally containing more myonuclei and satellite cells than fast [26,43]. Mouse EDL has a near-uniform (99%) fast fibre type in adult [23], and this relative distribution of fast and slow fibres is established early, and does not change throughout postnatal development [24,25]. There is a shift from the neonatal MyHC isoform to types IIa/b/x during postnatal development, but IIx and IIb fibres have similar myonuclear domains in both mouse and rat [36,44]. Therefore, the maturation of the myofibres to their adult fibre type, and near uniform fast fibre type composition during the period of study, both mean that expressing myonuclei and satellite cell numbers per EDL myofibre should not mask any major differences between muscle fibre types.

Since muscle satellite cells are the source of myonuclei postnatally [8], we also examined their number during the postnatal growth period using our two complementary mouse models. There are several useful markers for identifying satellite cells [45], but one of the most convenient is the expression of nuclear-localised β-galactosidase from the targeted *Myf5 *allele of *Myf5*^*nlacZ*/+ ^mice [21,46]. In contrast, the *3F-nlacZ-E *transgenic mouse has robust *nlacZ *expression in myonuclei, allowing the quantification of satellite cells by locating and counting DAPI-positive nuclei without β-galactosidase activity on isolated myofibres [21]. Since there is debate about whether certain antigens are expressed in all satellite cells [45,47], use of *3F-nlacZ-E *mice allows the entire satellite cell population to be identified, regardless of an antigen's expression domain. Importantly, counting either β-galactosidase-positive nuclei on myofibres from *Myf5*^*nlacZ*/+ ^mice, or non-*nlacZ *expressing nuclei on myofibres from *3F-nlacZ-E *mice using DAPI, gave similar results. Satellite cell number per myofibre was highest at the earliest age analysed, with ~14 satellite cells per myofibre at P6/7. There was a linear fall in satellite cell numbers to ~9 at P14, until the adult number of ~5 was reached by P21.

When satellite cell numbers are expressed as a percentage of total nuclei, their decrease is exaggerated by the rapid increase in myonuclear number (initially ~13 per day). Ontell reported that satellite cells account for 11% of sublaminal nuclei at P14, which decreases to 5% by P56 [4]. We found this to be closer to 4.6% at P14, falling to 2.3% by P56. Therefore while both studies report an approximate halving of the satellite cell pool during this period, the electron microscope studies appear to overestimate the number of satellite cells when using myonuclei per section as the denominator. It is interesting to note though, that our satellite cell and myonuclei data, projected back to birth, produce estimates that satellite cells would account for 31-35% of total sublaminal nuclei, the same range previously reported for mouse peroneus longus muscle at birth using electron microscopy [12].

Associating satellite cell and myonuclear number uncovered an interesting correlation: in the early postnatal period (before P9), the number of satellite cells per fibre is similar to the rate of myonuclear addition each day. There were ~13 satellite cells per myofibre until P9, when the mean addition of myonuclei was 13.6 per day. Since the satellite cell number is relatively stable over this period, it implies that most satellite cells are undergoing asymmetric divisions, each giving rise to both a myonucleus and a new satellite cell, as first suggested by Moss and Leblond in 1971 [8]. Between P14 and P21, the available number of satellite cells decreases from ~10 to ~5 per myofibre, and the rate of myonuclear accumulation decreases to 5.5 nuclei per day. While some satellite cells are clearly lost, most must still be producing both differentiated progeny and also undergoing self-renewal to maintain their own population.

Each satellite cell producing a single myonucleus and self-renewing each day indicates a cell cycle time of approximately 24 hours. It is a crucial to know however, how many of these satellite cells are actually proliferating? Pulsing with BrdU twice daily between P3 and P10, revealed that 78% of satellite cells in mouse EDL were labelled at P10, so had undergone at least one cell division during this period [15]. A similar proportion of satellite cells (80%) were also readily labelled by extended pulsing in growing rat muscle [14]. From this, we estimate an *in vivo *proliferation rate with a cell cycle doubling time of approximately 19 hours during the first two postnatal weeks, slowing to ~48 hours by P21. In adult, the estimated cell cycle doubling time is 1228 hours, i.e. the satellite cells have become quiescent as expected [17]. Combined BrdU and [^3^H]thymidine pulsing has been used to calculate a cell cycle time of 32 hours in growing rats *in vivo *[14]. Moss and Leblond [8] reported that the number of satellite cells that incorporated [^3^H]thymidine doubled within 24 hours of labelling and that those labelled cells contributed to myonuclei within 18 to 24 hours, indicating a significantly shorter cell cycle time. Growth dynamics are different between rats and mice though, with the EDL adding myonuclei until P60 in rat [2,14], so it would not be unexpected if cell cycle time in growing mouse was significantly shorter. For adult, Bischoff has calculated cell cycle times for mouse satellite cells *in vitro *at 12 hours [48], whereas we have previously calculated about 18 hours [26].

Interestingly, it has recently been shown that the transcription factor Pax7 is essential for satellite cell function up to ~P21 [49], which coincides with the age that myofibres cease to grow by the addition of myonuclei supplied by satellite cells, and a population of mitotically quiescent satellite cells is established. Around P21 is also when the chronic cycles of degeneration and regeneration begin in the *mdx *mouse model of Duchenne muscular dystrophy [50]. Speculatively, the presence of actively dividing satellite cells before P21 may permit the rapid repair of damaged myofibres, so ameliorating the dystrophic phenotype at this time. However, muscle damage occurring after P21 must first elicit satellite cell activation from quiescence, which may delay the repair process, so allowing myofibre degeneration to occur.

## Conclusions

Since the total myofibre content of EDL does not change during the first three weeks of postnatal life, muscle growth is achieved by myofibre hypertrophy, which is accompanied by the addition of significant amounts of new myonuclei (~5 fold increase). Despite this increase in myonuclear number during this period, the myonuclear domain still doubles in size. The number of satellite cells per myofibre is similar to the daily myonuclear accretion rate until P21, when the adult configuration of myonuclei and satellite cells is established. The significant myofibre hypertrophy that continues from P21 to adulthood therefore, occurs without the net addition of myonuclei, so results in a large (3-fold) expansion of the myonuclear domain.

## Methods

### Myofibre isolation

Animal husbandry, breeding and experimental procedures were passed by the Ethical Review Process Committee of King's College London, and were carried out in accordance with British law under the provisions of the Animals (Scientific Procedures) Act 1986. Postnatal mouse age (P) was calculated with the day of birth being designated as P0. Age-matched male and female *Myf5*^*nlacZ*/+ ^[20,21,46] and *3F-nlacZ-E *[21,28] mice were killed by cervical dislocation and the EDL muscles were carefully dissected. Muscles were digested in 0.2% Collagenase Type 1/DMEM (Sigma); individual myofibres were dissociated by gently passing through Pasteur pipettes with different sized apertures and then abundantly washed, as described in detail elsewhere [51,52]. Since damaged myofibres partially or completely hypercontract in culture, they are easy to distinguish from the viable, intact myofibres with tapered/sculptured ends that were selected for fixation in 4% paraformaldehyde/PBS (Sigma) for 6 minutes.

### Histochemistry

To visualize β-galactosidase activity, fixed myofibres were incubated overnight (*Myf5*^*nlacZ*/+^) or for 2 hours (*3F-nlacZ-E*) at 37°C in X-gal solution (4 mM potassium ferrocyanide, 4 mM potassium ferricyanide, 2 mM MgCl_2_, 400 μg/ml X-gal and 0.02% NP40 in PBS) and thoroughly washed in PBS before mounting in Vectashield mounting medium (Vector Laboratories, Inc.) containing 100 ng/ml 4,6-diamidino-2-phenylindole (DAPI).

### Counting myonuclei and satellite cells

Total myofibre nuclei (DAPI-positive plus β-galactosidase-positive), total myonuclei per myofibre (DAPI-positive minus β-galactosidase-positive for *Myf5*^*nlacZ*/+ ^mice, and just β-galactosidase-positive for *3F-nlacZ-E *mice) and total satellite cells per myofibre (β-galactosidase-positive for *Myf5*^*nlacZ*/+ ^mice, but DAPI-positive and β-galactosidase-negative for *3F-nlacZ-E *mice) were counted using a Zeiss Axiophot 200 M microscope. Six or more mice were used for P3, P7, P14, P21, and P56, comprising at least three each of *Myf5*^*nlacZ*/+ ^and *3F-nlacZ-E*. For other time points (P4, P5, P6, P9, P17, P28 and 10 months of age), at least two *Myf5*^*nlacZ*/+ ^mice were analysed at each stage.

### Measuring myofibre and sarcomere length

For measurement of myofibre length, images were captured on a Leica MZ16F dissecting microscope using an Olympus DP70 camera, and analysed using ImageJ software (NIH - version 1.42q). Myofibre length was measured from between 19 - 41 isolated myofibres each from at least 3 mice per time point. For measurement of sarcomere length, brightfield images along myofibres were taken on a Zeiss Axiophot 200 M microscope and analysed using NIH Image J software http://rsbweb.nih.gov/ij/. Over 200 sarcomeres from at least 5 myofibres were measured from each of 3 mice per time point.

### Muscle cryosectioning and staining

The muscular anterior compartment (tibialis anterior (TA) and EDL muscles) of at least 3 *Myf5*^*nlacZ*/+ ^mice each at P7, P14, P21 and P56 were removed, the proximal extremity fixed on a piece of cork with tragacanth gum and frozen in isopentane cooled by liquid nitrogen. Samples were stored at -80°C and serial 10 μm cross-sections then cut with a cryostat, collected throughout the entire EDL muscle and transferred to 3-aminopropyl-trietoxylane (Sigma, A3648) coated glass coverslips.

On cryosections from the mid-belly level of the EDL, haematoxylin-eosin or immunostaining for laminin were performed to delimit myofibres for counting. Primary antibody used was a polyclonal rabbit anti-Laminin IgG (Sigma L-9393, 1:200) visualized using Alexa Fluor 594, goat anti-rabbit IgG (Invitrogen A11037, 1:200). All antibodies were diluted in Phosphate Buffered Saline (PBS) and sections were first incubated with the primary antibody in a humidified closed incubation chamber at room temperature for 1 h. After 3 rinses with PBS, the primary antibody was localised with the secondary antibody applied for 1 h, at room temperature. Sections were mounted with Vectashield mounting medium (Vector Laboratories, Inc.) containing 100 ng/ml DAPI and examined using a Zeiss Axiophot 200 M microscope. To identify satellite cells, cryosections of EDL from *Myf5*^*nlacZ*/+ ^were fixed and incubated in X-gal, before being counterstained with eosin to mark myofibres. All nuclei containing blue X-gal reaction product were then counted.

### Determination of myofibre number and cross-sectional area

Images were made of entire cross-sections of laminin immunostained or haematoxylin-eosin stained mid-belly EDL muscles from 3 mice at each age and total myofibre number determined. Cross-sectional area was determined by measuring at least 300 myofibres in total from at least 3 mice per time point using images from cryosections of mid-belly level EDL. Measurements were obtained using NIH Image J software http://rsbweb.nih.gov/ij/.

### Data Analysis

Data were processed using Microsoft Excel and statistical analyses performed using SPSS v16.0. Data are presented as mean ± SEM. Comparisons of means were made using ANOVA with Tukey's HSD post-hoc testing and regression analysis was performed for goodness-of-fit. All data analysed by ANOVA satisfied homogeneity of variance, as defined by Levene's Statistic (p < 0.01). Significance was determined as at least p < 0.05. Some data is also presented in graph form to better illustrate the regression analysis.

## Authors' contributions

RBW participated in the design of the study, carried out the majority of the experimental work, performed the statistical analysis and helped draft the manuscript. A-SB carried out the analysis on number of myofibres and satellite cells on muscle sections. VFG measured myofibre and sarcomere length and cross-sectional area, and contributed to writing the manuscript. PSZ conceived the study, participated in its design and coordination, and drafted the manuscript. All authors read and approved the final manuscript.
